# Plasma VEGF and Leptin Values in Patients With Gastric Intestinal Metaplasia and Metabolic Syndrome

**DOI:** 10.3389/fonc.2022.905168

**Published:** 2022-05-31

**Authors:** George Pappas-Gogos, Kostas Tepelenis, Anna Goussia, Constantinos Tellis, Fotis Fousekis, Georgios K. Glantzounis, Konstantinos Vlachos

**Affiliations:** ^1^ Department of Surgery, Ioannina University Hospital, Ioannina, Greece; ^2^ Department of Pathology, Ioannina University Hospital, Ioannina, Greece; ^3^ School of Medicine, University of Ioannina, Ioannina, Greece; ^4^ Laboratory of Biochemistry, Department of Chemistry, University of Ioannina, Ioannina, Greece; ^5^ Department of Gastroenterology, Ioannina University Hospital, Ioannina, Greece

**Keywords:** plasma, VEGF, leptin, gastric intestinal metaplasia, metabolic syndrome

## Abstract

Intestinal metaplasia of the stomach (IM) is considered a pre-cancerous lesion and is a potential precursor to adenocarcinoma. Metabolic syndrome (MetS) has been associated with lesions to the gastrointestinal tract such as the risk of developing Barett esophagus. Vascular endothelial growth factor and leptin have been associated with either gastrointestinal tract carcinogenesis or MetS. In this context, this study was designed to analyze plasma levels of VEGF and leptin in patients with IM and MetS. Four groups of 137 participants (a control group and three patient groups, IM, MetS and IM- MetS) were created. Inclusion criteria for the presence of IM were endoscopic findings and histological confirmation, while for MetS the ATP III and IDF guidelines. Levels of plasma vascular endothelial growth factor (VEGF) and leptin (Leptin) were determined. VEGF levels were increased in IM (IM vs Control, p=0,011) and IM-MetS groups (IM-MetS vs Control, p <0.001 and IM-MetS vs MetS, p=0.001). Leptin levels were found to be increased in the MetS group (MetS vs. Control, p <0.001 and MetS vs IM, p <0.001) and in IM-MetS (IM-MetS vs Control, p = 0.002, IM-MetS vs IM, p=0.033). Patients with intestinal metaplasia and metabolic syndrome (I M - Me t S g r o u p) have elevated levels of VEGF, while leptin levels were associated predominantly with MetS and not with IM.

## Introduction

IM is considered a precancerous lesion and is associated with increased risk of developing gastric cancer. Endoscopic monitoring has been proposed to check the lesion of endangered populations (1). However, there are no widely accepted guidelines on IM management. Recently, the European Endoscopic Society as well as other European academic institutes have developed documented guidelines for the management of patients with IM. These guidelines emphasize the risk of cancer and the need for staging in high-grade dysplasia. Risk factors for IM include Helicobacter pylori infection, high NaCl intake, smoking, alcohol consumption and chronic bile reflux (2). Based on the available data, 45% of patients with gastric adenocarcinoma of the cardia has residual IM, supporting the idea that IM is a particularly significant precursor to its development (3). Chronic inflammation has been recognized as an important risk factor for the carcinogenesis of the gastrointestinal tract by causing cell mutations and promoting malignant transformation in the normal epithelial cells (4). Obesity, especially abdominal obesity, is associated with peripheral tissue insulin resistance and fatty acid metabolism, often leading to type 2 diabetes mellitus (SD2). Insulin resistance, hyperinsulinemia, hyperglycemia and the production of adipokines can also lead to endothelial dysfunction, disturbance of the lipid profile, hypertension and vascular inflammation, which promote the development of atherosclerotic cardiovascular disease.

In patients with coexistence of metabolic risk factors for SD2 and cardiovascular disease (abdominal obesity, hyperglycemia, dyslipidemia and hypertension) the existence of “metabolic syndrome” (MetS) has been suggested (5, 6). MetS represents a proinflammatory and prothrombotic condition associated with elevated levels of CRP, IL-6, and plasminogen activator inhibitor (PAI) 1. Some of those inflammatory and prothombotic markers which are associated with increased risk of cardiovascular disease and SD2 represent only a part of the association between MetS and cardiovascular mortality. Thus, the value of calculating some markers in the follow-up of patients with MetS is unknown (7, 8).

Studies that have analyzed possible association of MetS and gastrointestinal tract lesions reported that patients with MetS are almost twice as likely to develop Barett esophagus (BE). The prevalence of MetS in relation to the development of BE and especially the relationship between the length of the lesion and the changes in the levels of leptin, insulin and pro-inflammatory markers, shows that the changes caused during the development of BE are continuous and are affected by the metabolic changes caused by adipokines and cytokines (9). On the other hand, the study of the association between MetS and the development of IM requires prospective studies. It has been demonstrated that insulin may potentiate the mitogenic effect of EGF, which in turn activates pathways such as the Ras/mitogen-activated kinase, and promotes also the proliferation of the gastric mucosa cells (10). Other studies have reported strong association between obesity, SD2 and the development of IM, as well as of gastric cancer (11–13).

Based on these observations we aimed to study the association between MetS and IM by analyzing markers such as VEGF and leptin which involved in both pathological conditions.

VEGF represents one of the most important growth and survival factors for the endothelium. VEGF plays an important role in the regulation of angiogenesis (14, 15). VEGF is a glycoprotein bound to heparin and secreted as a 45 kDa homodimer. Even larger types of cells and not only endothelial cells secrete VEGF. Because the newly discovered VEGF, VEGF-A, increases vascular permeability, it was also known as the vascular permeability factor. In addition, VEGF causes vasodilation partly through stimulation of nitric oxide synthetase in endothelial cells (16). VEGF can also stimulate cell migration and inhibit apoptosis (17). Inducible hypoxia, in particular, characterizes VEGF-A against other members of the VEGF family and other angiogenic agents. The transcription of VEGF into normoxia is activated by many oncogenes including K-ras and several transmembrane tyrosine kinases such as the epidermal growth factor receptor (EGFR) and ErbB2 (16–20). These pathways constitute an important upward adjustment of VEGF-A in tumors compared to normal tissues and often have prognostic significance (21).

Carcinogenesis is a multistage process in which angiogenesis represents an essential step. Cancer growth, invasion and metastases depend from oxygen, nutritive substances, and growth factors delivered by angiogenesis. VEGF is one of the most important growth factors, and essential for tumor angiogenesis (22). Several studies have shown the association of VEGF with MetS or its components. Results of a meta-analysis reported a strong association of increase in VEGFs expression in subjects with MetS, hyperglycemia, hypertriglyceridemia and hypertension (23).

Leptin is a hormone produced mainly by fat cells and helps regulate energy balance by preventing hunger, and its action was found in receptors in the arcuate nucleus of the hypothalamus. In obesity, there is a decreased sensitivity to leptin (similar to insulin resistance in type 2 diabetes), resulting in the inability to detect satiety even in large energy reserves and high leptin levels (24). Although the regulation of fat reserves is considered to be the main function of leptin, it also plays a role in other physiological processes, as evidenced by the many sites of its composition, in addition to fat cells, and the many types of cells next to hypothalamic cells that have leptin receptors, any of these additional functions have not yet been identified (25). The primary function is to regulate the mass of adipose tissue through the central affects caused by fasting, energy consumption, physical exercise and energy balance. Its secondary functions are the modulation of energy expenditure, the metabolism between the fetus and the mother, it acts as a permissive factor during adolescence, as well as an activator of immune cells, an activator of pancreatic beta cells and as a growth factor. While leptin is associated with body fat mass, the size of individual fat cells and overconsumption, it is interesting that it is not affected by exercise (compared to IL-6, which is released in response to muscle contractions). Thus, it is speculated that leptin responds specifically to inflammation that comes from adipose tissue. Leptin is a pro-angiogenic, pro-inflammatory and mitogenic agent, the actions of which are enhanced through crossovers with cytokines of the IL-1 family in cancer (26, 27). Furthermore, it has been showed the role of leptin/leptin receptors in modulating T cell activity. Exogenous leptin may promote angiogenesis by increasing levels of VEGF (28–30).

## Materials and Methods

### Inclusion, Preparation and Randomization of Patients

The study received approval from the ethics committee of GNFiliaton (1, 21/1/2016). The participants in the study (patients and controls) were selected from among those who underwent gastroscopy in the Gastroenterology department. According to the inclusion/exclusion criteria, those who consented to take part in the study were allocated on the basis of the findings from the clinical examination, gastroscopy, as well as laboratory and histological analyses. Those who did not have any pathological findings in the clinical/laboratory examination constituted the control group. Patients were divided in three groups. MetS (patients with MetS only), IM (patients with IM without MetS) and IM-MetS (patients with IM and MetS).

Inclusion criteria for MetS (ATPIII and IDF) were abdominal obesity (AO) with an average diameter of ≥102 cm in men and ≥88 cm in women. Serum triglycerides (TRG) ≥150 mg/dL (1.7mmol/L), or treatment for elevated triglycerides. Serum HDL<40 mg/dL (1 mmol/L) in men and <50 mg/dL (1.3mmol/L) in women, or receiving treatment for decreased HDL. Blood pressure (BP) ≥130/85mmHg, or receiving treatment for arterial hypertension. Fasting glucose (Glu) levels ≥100mg/dL (5.6mmol/L), or receiving treatment for hyperglycemia.

Study population included either subjects non infected, or patients infected by H. pylori, since H. Pylori has a strong association with the development of IM. However, inclusion criteria for IM was endoscopic findings and histological confirmation.

Exclusion criteria were chronic inflammatory diseases (autoimmune diseases, inflammatory diseases of the colon), history of angiopathy (the exception was diabetics), active smokers, and PPI’s treatment. Blood sampling was carried out by an operator who had not been informed of the study. Similarly, the staff of the laboratory that carried out the analyses did not have access to the study.

### Blood Sampling and Preservation

Blood sampling were performed before gastroscopy. All samples were taken from peripheral veins. Immediately after the blood sampling the blood was centrifuged (4000rpm for 5min) and the supernatant was stored in cryovials at -80°C.

### Determination of Levels of VEGF

VEGF (pg/ml) levels in the patients’ plasma were determined by enzyme immunoassay (Human VEGF Quantikine ELISA Kit, R&D Systems, Inc., Minneapolis, MN, USA), following the manufacturer’s instructions.

### Determination of Leptin Levels (Leptin)

Leptin levels (ng/ml) in the plasma of patients were identified in the method of enzyme immunoassay (Human Leptin ELISA, Clinical Range, BioVendor-Laboratorni medicina.s.Brno, Czech Republic) following the manufacturer’s guidelines.

### Statistical Analysis

It was estimated that at a p ≤ 0.05, to determine a 10% difference between the two groups, in terms of levels of markers, a sample of a total of 100 patients (25 patients for each group) would have a validity of 90% (GPower 3.1). A total of 137 patients were included in the study, so that a percentage of ~10% could be excluded. Statistical analysis was carried out with Statistical Package for the Social Sciences (SPSS) ver.19.0 (IBM). The regularity of the distribution of quantitative variables was analyzed by the Kolmogorov–Smirnov test. Since the levels of the variables studied had a normal distribution, they were expressed as mean ± standard deviation (SD).

The differences between the groups were analyzed with one-way ANOVA and the *post hoc* analysis was carried out with the Bonferroni correction. Patients’ characteristics, were analyzed with chi-square (gender) and one-way ANOVA (age, AO, BP, Glu). All analyses were two-sided, while a p < 0.05 were considered statistically significant.

## Results

### Patients’ Characteristics

Among the groups, no statistically significant differences were found in terms of gender and age (**Table 1**).

**Table 1:** Study participants’ characteristics.

Characteristics	Control	MetS	IM	IM- MetS	*p* value
Gender, Male, n (%)	28 (20.4)	17 (12.4)	10 (7.3)	15 (10.9)	0.10^*^
Age, years, (SD)	59.5 (14.2)	58.9 (10.6)	54.2 (12.5)	49.8 (13.2)	0.20^**^

^*^Chi-square; ^**^One-way ANOVA.

### Analysis of the Characteristics of MetS

Analysis of MetS-related characteristics showed statistically significant differences between groups (**Table 2**), regarding AO (p<0,001), BP (p<0,001), Glu (<0,001), TRG (p=0,007) and HDL levels (p<0,001).

**Table 2:** Comparison of MetS characteristics between the groups.

	Groups	N	Mean ± SD	95%CI		*p* value^*^
				Lower	Upper	
AO	Control	49	90.65 ± 5.37	88.13	93.16	<0.001
	MetS	33	106.66 ± 7.41	100.96	112.36	
	IM	27	87.28 ± 6.12	81.61	92.95	
	IM- MetS	28	104.37 ± 6.67	98.79	109.95	
BP	Control	49	117.66 ± 5.62	115.67	119.66	<0.001
	MetS	33	136.88 ± 8.15	132.69	141.07	
	IM	27	122.72 ± 9.24	116.51	128.93	
	IM- MetS	28	137.33 ± 4.61	134.39	140.26	
Glu	Control	49	92.68 ± 31.84	79.53	105.82	<0.001
	MetS	33	189.25 ± 39.51	168.19	210.30	
	IM	27	90.23 ± 16.49	79.19	101.35	
	IM- MetS	28	168.41 ± 32.89	147.51	189.31	
TRG	Control	49	100.45 ± 81.42	71.58	129.32	0.007
	MetS	33	177.88 ± 89.39	131.91	223.84	
	IM	27	137.90 ± 52.61	102.56	173.25	
	IM- MetS	28	154.25 ± 47.74	123.91	184.58	
HDL	Control	49	65.27 ± 18.83	58.59	71.94	<0.001
	MetS	33	37.11 ± 12.56	30.64	43.57	
	IM	27	62.54 ± 22.54	47.39	77.69	
	IM- MetS	28	35.33 ± 4.79	32.28	38.38	

^*^Statistical significance between the groups; One-way ANOVA.

### Analysis of VEGF Levels

VEGF levels were found to have statistically significant differences between groups (p<0,001) (**Table 3**). In-group analysis (**Table 4**) showed significantly elevated levels of VEGF in the patients with IM as well as IM and MetS. More specifically, IM patients had significantly increased levels of VEGF compared to controls (p=0.011), while even more increased were the levels of patients with IM-MetS, compared to controls (p<0.001).

**Table 3:** Analysis of VEGF and Leptin values between the groups.

	Groups	N	Mean ± SD	95% CI		*p* value^*^
				Lower	Upper	
VEGF	Control	49	51.20 ± 32.85	36.24	66.15	<0.001
	MetS	33	58.8 ± 32.85	63.28	110.42	
	IM	27	123.50 ± 71.23	68.74	178.25	
	IM- MetS	28	181.15 ± 82.02	129.03	233.26	
LEPTIN	Control	49	2.21 ± 2.99	0.76	3.65	<0.001
	MetS	33	17.60 ± 11.10	11.19	24.01	
	IM	27	3.37 ± 3.91	0.37	6.38	
	IM- MetS	28	13.66 ± 10.34	6.26	21.05	

^*^Statistical significance between the groups; One-way ANOVA.

**Table 4:** In-group analysis of VEGF and Leptin values.

Dependent Variable	Group comparisons		Mean Difference	95% CI		*p* value^*^
				Lower	Upper	
VEGF	Control	MetS	-35.65	-87.80	16.48	0.131
		IM	-72.30	-132.51	-12.08	0.011
		IM- MetS	-129.95	-184.63	-75.26	<0.001
	MetS	IM	-36.64	-101.21	27.92	0.098
		IM- MetS	-94.29	-153.74	-34.83	0.001
	IM	IM- MetS	-57.65	-124.29	8.91	0.067
LEPTIN	Control	MetS	-15.39	-22.86	-7.93	<0.001
		IM	-1.16	-9.74	7.41	0.127
		IM- MetS	-11.44	-19.73	-3.16	0.002
	MetS	IM	14.22	5,17	23.28	<0.001
		IM- MetS	3.94	-4.52	12.72	0.094
	IM	IM- MetS	-10.28	-20.02	-0.54	0.033

^*^One-way ANOVA, Bonferroni post hoc multiple comparisons.

**Figure 1:**
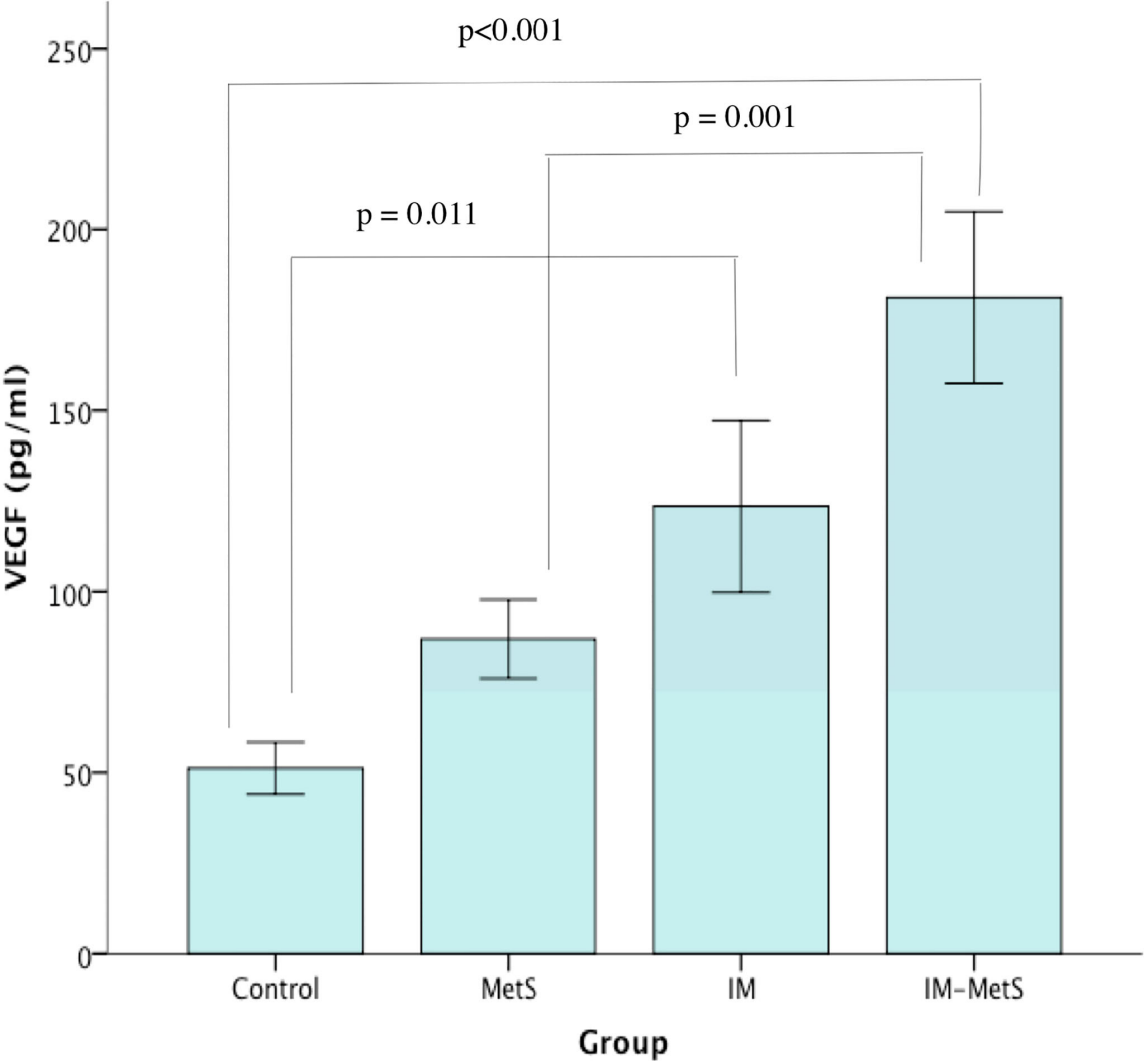
VEGF levels in patients and control.

Also, VEGF levels of IM-MetS were significantly higher compared to MetS (p=0.001). It appears that VEGF levels are affected by the presence of IM primarily (**Figure 1**).

### Analysis of Leptin Levels

**Figure 2:**
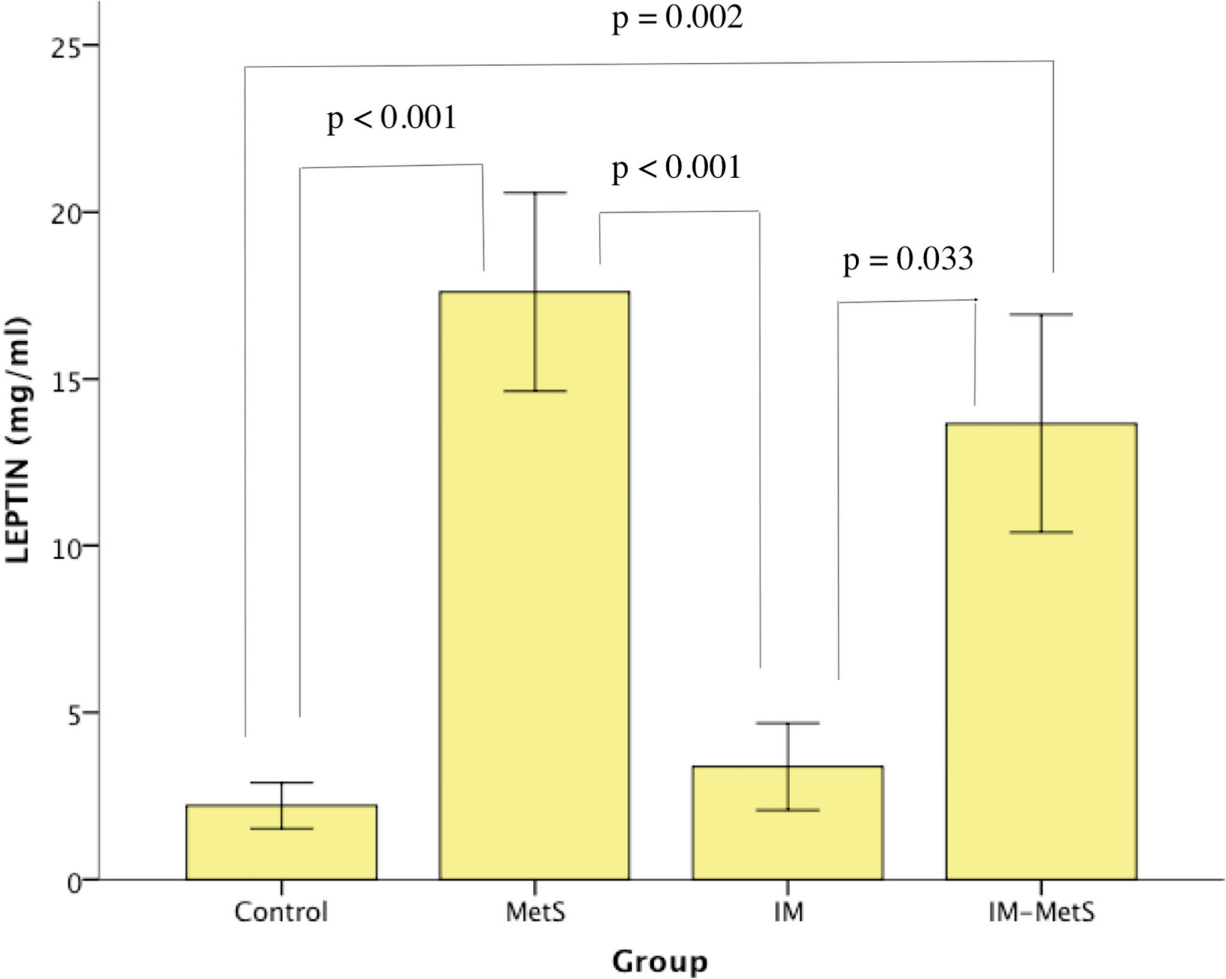
Leptin levels in patients and controls.

Leptin levels were found to have statistically significant differences between groups (p<0,001) (**Table 3**). In-group comparisons (**Table 4**) revealed that patients with MetS, as well as patients with IM-MetS, showed the higher increase in leptin levels. More specifically, IM-MetS showed a significant increase compared to the control group (p<0,001 and p=0,002 respectively). This increase was also significant compared to the levels of IM patients (p<0,001 and p=0,033 respectively). It seems that leptin levels are affected by the existence of metabolic syndrome (**Figure 2**) and not by the presence of intestinal metaplasia.

## Discussion

It has been demonstrated that patients with MetS are at a higher risk of developing BE even without showing symptoms of reflux (31). There is a strong correlation between MetS, “central type” obesity, and BE. The prevalence of MetS in cohort BE, and in particular the relationship between the extent of BE and the change in leptin, insulin and other pro inflammatory markers in plasma, indicates that changes in the mucosa of patients with BE can be continuous and affected by metabolic changes in antacids and cytokines (32). Regarding IM, it has been reported that obesity and SD2, which represent two components of MetS, are significantly associated with the development of IM (11–13, 33).

In this study, both groups with IM and those with IM-MetS showed a greater increase of VEGF in plasma. VEGF has been found elevated in histological preparations of patients with stomach cancer, an increase which was parallel to the expression of COX-2 (34). In particular, its levels have been found elevated in patients with intestinal type gastric cancer, lesion which is associated with IM. An increase in its levels has also been reported in patients with esophagitis and in patients with Barrett’s esophagus (35, 36). Regarding the correlation of MetS with the VEGF, studies have shown that VEGF levels are related to MetS components. Thus, patients with SD2 appear to have elevated levels, in contrast to obese people who have seen an increase in VEGF-B and VEGF-C levels rather than VEGF-A. On the other hand, dyslipidemic patients experienced increased levels of VEGF (37). In the present study, where VEGF was identified in patients with MetS genetics, and not in the individual components of the syndrome, no increase in levels was found in relation to the control group, nor to the other groups. In the present study, it has been taken into consideration the analysis of VEGF in patients with MetS in general but not in the distinctive components of the syndrome.

The determination of plasma leptin levels showed a significant increase in levels in patients with MetS and IM-MetS. Regarding the increased levels of leptin in patients with MetS there are several reports confirming their increase, which is mainly related to the mass of adipose tissue, while on the other hand its levels are also related to insulin resistance (38).

In our study, patients with IM, like those with IM-MetS, did not experience elevated leptin levels. Reports from the international literature regarding leptin levels in IM patients are contradictory. It appears that IM is not related to plasma leptin levels, as patients with BE and IM, or with IM, did not experience increased levels (38, 39). However, studies of histological preparations in patients with IM and patients with gastric cancer report an increase in expression and levels of gastric leptin (40–42). The association of leptin with the development of IM in patients with or without MetS remains to be clarified.

This is the first study which analyzes these markers in patients with MetS and IM, as well as with both MetS and IM. We believe that the results, although this is not a randomized trial, might contribute to conception and design of studies, in order to further elucidate the mechanisms, as well as the association between MetS and IM, especially since either MetS or IM are associated with GI carcinogenesis.

This study has some limitations which must be acknowledged. Firstly, the analyses of plasma VEGF and leptin levels concerned MetS in general, but not each component of it. It would be very interesting to study the possible contribution of each component of MetS in the development of IM. Moreover, it could be interesting to study the possible association of each form of VEGF with MetS and the development of IM.

## Data Availability Statement

The datasets presented in this study can be found in online repositories. The names of the repository/repositories and accession number(s) can be found in the article/supplementary material.

## Ethics Statement

The studies involving human participants were reviewed and approved by Filiates General Hospital ethical committee. The patients/participants provided their written informed consent to participate in this study.

## Author Contributions

Conceptualization, GP-G, FF, and GG. Methodology, GP-G, CT, FF, AG, KT, KV, and GG. Statistics, GP-G, FF, and KT. Investigation, GP-G, FF, GG, and KV. Data curation, AG, CT, GP-G, GG, and KV. Writing—original draft preparation: GP-G, KT, and KV. Writing—review and editing: all authors. Supervision, KV and GG. All authors have read and agreed to the published version of the manuscript.

## Conflict of Interest

The authors declare that the research was conducted in the absence of any commercial or financial relationships that could be construed as a potential conflict of interest.

## Publisher’s Note

All claims expressed in this article are solely those of the authors and do not necessarily represent those of their affiliated organizations, or those of the publisher, the editors and the reviewers. Any product that may be evaluated in this article, or claim that may be made by its manufacturer, is not guaranteed or endorsed by the publisher.
